# Supplementary material for: A transcriptional network required for bradyzoite development in Toxoplasma gondii is dispensable for recrudescent disease
Source: Nat Commun. 2023 Sep 28;14:6078. doi: 10.1038/s41467-023-40948-w (PMC10539341; doi:10.1038/s41467-023-40948-w)
Supplement: Supplementary file 1 — Supplementary Information [file 41467_2023_40948_MOESM1_ESM.pdf]

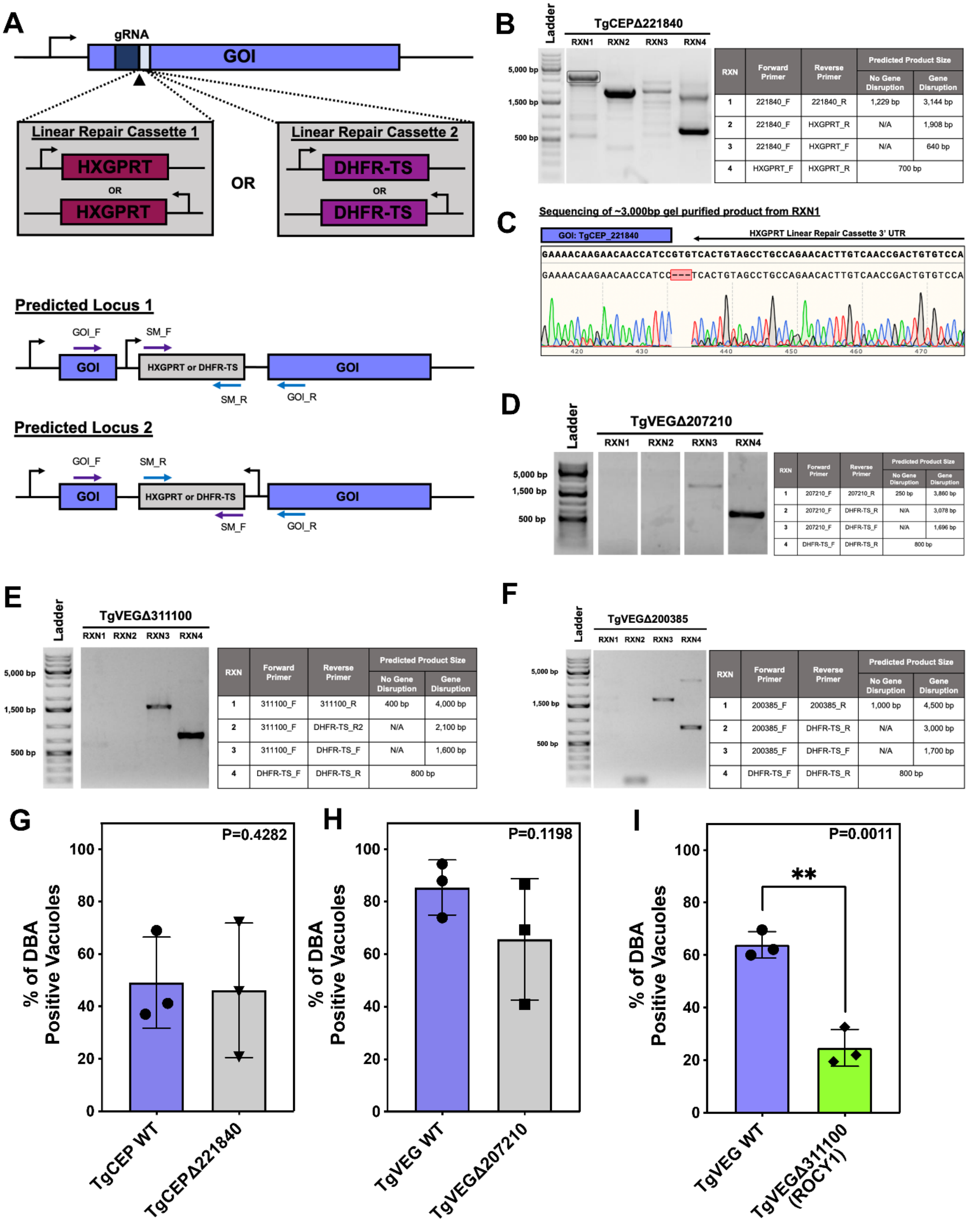

Figure S1. Generation of knockout of high priority candidate genes using CRISPR/Cas9. A) Schematic of CRISPR/Cas9 knockout and repair strategy and diagram of predicted loci of gene of interest (GOI) after incorporation of linear repair cassettes of selectable markers (SM) HXGPRT or DHFR-TS. Arrows indicate general location of primers. B) Gel image and PCR reactions used to identify disruption of the TgCEP_221840 locus. C) Sanger sequencing data of of gel purified ~3,000 bp band from RXN1 sequenced with 221840_F primer. D) Gel image and PCR reactions used to identify disruption of the TgVEG_207210 locus. E) Gel image and PCR reactions used to identify disruption of the TgVEG_311100 (*ROCY1*) locus. F) Gel image and PCR reactions used to identify disruption of the TgVEG_200385 (*BFD1*) locus. G, H and I) Percent DBA+ cyst

formation for G) TgCEPΔ221840 H) TgVEGΔ207210 I) TgVEGΔ311100 (*ROCY1*) and passaged matched WT strains after 48 hours of alkaline pH induced stress. The mean and standard deviation (N=3 biological replicates) of the percentage of DBA positive vacuoles observed in 15 parasite containing fields of view is plotted. Statistical significance was determined using a Student's one-tailed t-test on arcsine transformed percentage data. Experiment performed a single time for each parasite line.

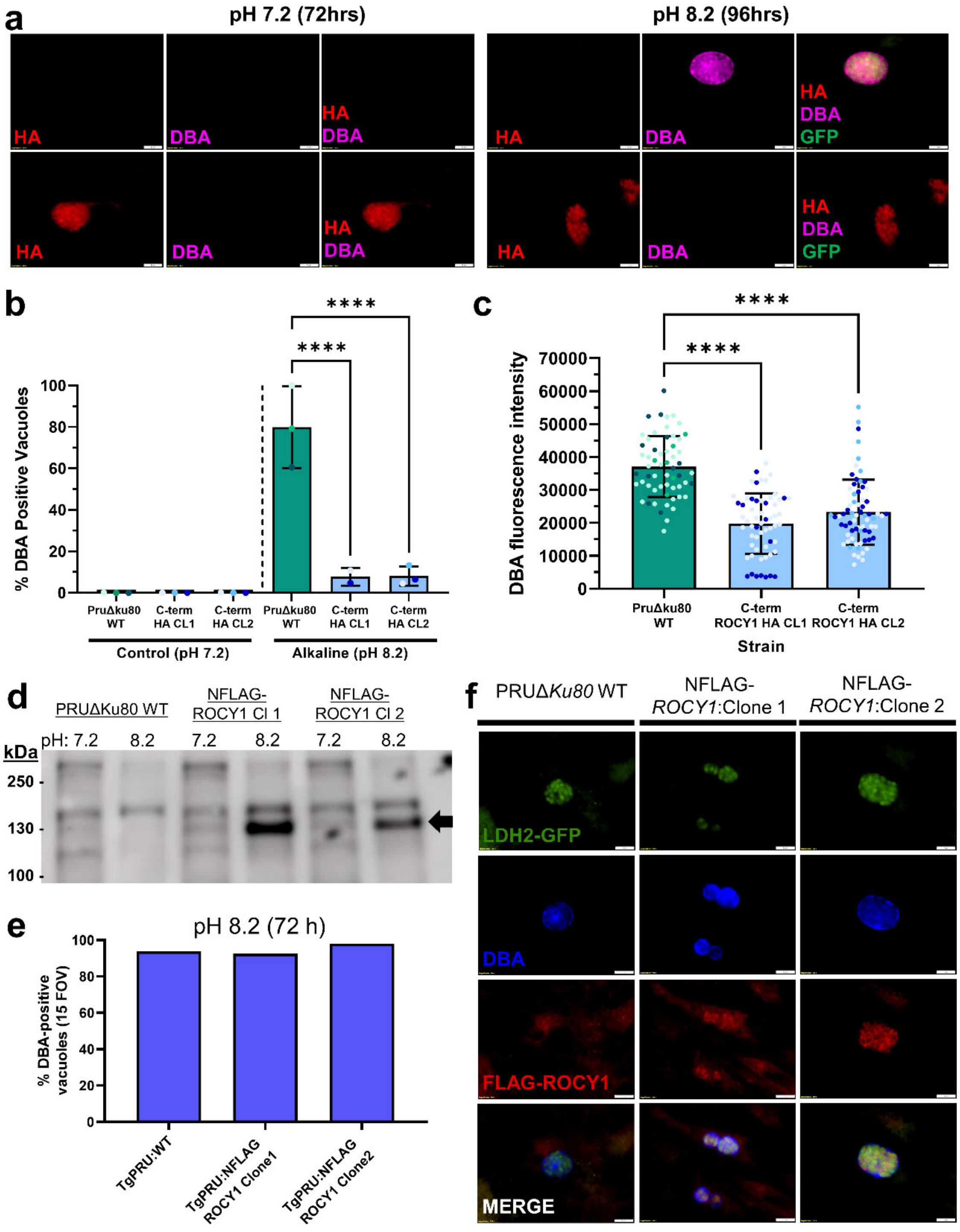

Figure S2: C-terminal and N-terminal tagging of *T. gondii ROCY1* has distinct impacts on gene function with respect to pH-induced cyst formation. A) HFFs were infected at an MOI of 0.3 with *T. gondii* (PruΔ*ku80* WT and PruΔ*ku80* C-terminally HA tagged *ROCY1*) and grown in pH 7.2 for 4 days or in pH 8.2 for 3 days, fixed and stained with an anti-HA and biotinylated DBA. B) Quantification of the percentage of DBA-positive cysts from the infection in A. Stress-induced cyst formation is markedly impaired in clones with a 3X C-terminal HA tag at

the ROCY1 locus. Mean +/- SD (N=3 biological replicates per condition) are shown and statistical significance was determined by one way ANOVA followed by Sidak's multiple comparison post test on arcsin transformed data. ****: P<0.0001. C) Detection and quantification of DBA fluorescent staining within parasite vacuoles (N=186 for WT, 128 for tagged clone 1 and 69 for tagged clone 2) using KNIME. Mean +/- SD and points are colored by the coverslip they were derived from (N=3 coverslips per condition and strain). Statistical significance was determined using one way ANOVA followed by Sidak's multiple comparison post-hoc test using individual vacuoles as biological replicates. (****=P < 0.0001, **=P ≤ 0.01) D) Indicated parasite lines harboring a single N-terminal Flag tag were grown under control or high pH conditions for 3 days and then harvested for western blotting. A band with an apparent molecular weight of ~130 KDa can be readily detected in high pH conditions only in the FLAG-tagged clones (arrow). This experiment was performed once. E) Quantification of cyst formation capacity in TgPRU:WT and the two N-terminally FLAG-tagged clones, showing that the N-terminal FLAG tag has no detectable impact on stress-induced cyst formation (in contrast to the impact of C-terminally tagging ROCY1). This experiment was replicated a single time (1 coverslip per parasite line). All scale bars: 10 μm. F) Representative images for PruΔ*ku80* WT, NFLAG ROCY1 Δ*ku80* Clone 1, and NFLAG ROCY1 Δ*ku80* Clone 2 after 3 days in alkaline switch conditions. Parasites endogenously express GFP driven by the LDH2 promoter, cysts were stained with biotinylated DBA and ROCY1 was localized using anti-FLAG antibodies. Detectable expression of NFLAG-ROCY1 can be seen localizing to perinuclear puncta, similar to that observed for C-terminally tagged parasites. Scale bar represents 10 μM. This experiment was performed once. Data available in Source Data File.

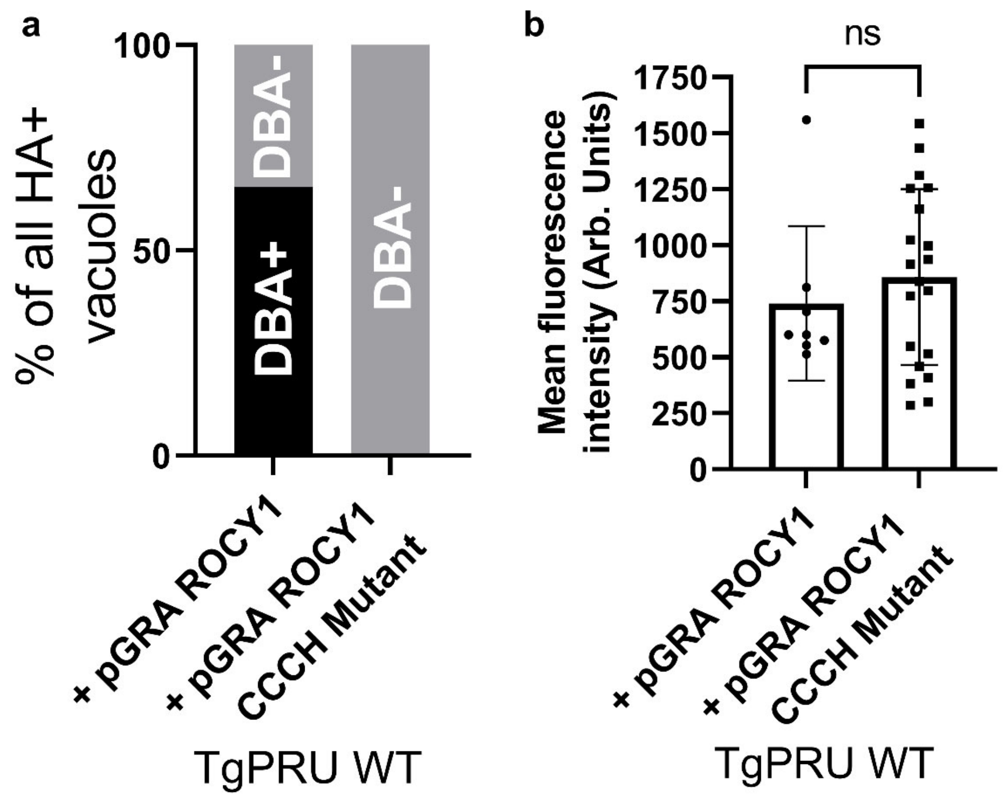

Figure S3: A) Quantification of the percentage of DBA-positive cysts among HA-positive vacuoles expressing the WT pGRA NHA-ROCY1 (N=26) or the CCCH mutant form of pGRA NHA-ROCY1 (N=25) under control (pH 7.2) conditions. The CCCH mutations ablated the ability of pGRA NHA-ROCY1 to induce cyst formation in the wild type PRU background. B) Quantitative immunofluorescence of background normalized mean HA-derived fluorescence intensity of TgPRU WT expressing either WT pGRA NHA-ROCY1 (N=8) or the CCCH mutant form of pGRA NHA-ROCY1 (N=20) showing similar staining intensity (Two-tailed Student's T-test *P*=0.4). This experiment was performed once. Data available in Source Data File.

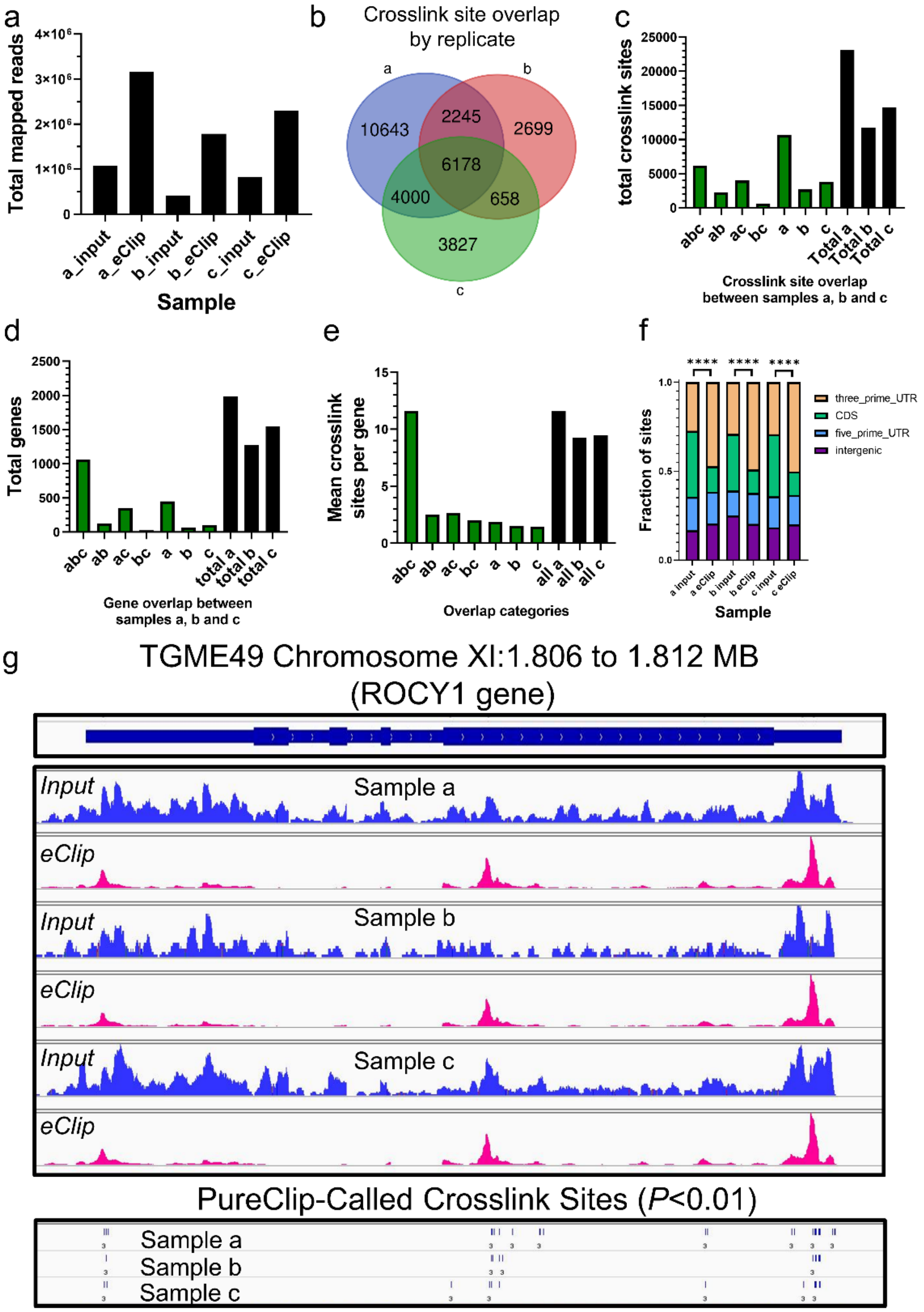

Figure S4: Analysis of 3 biologically independent eCLIP replicates using PureClip. A) Total mapped reads from each input and eCLIP sample. B) Overlap of sites between each of the 3 biological replicates at a significance level of P<0.01. Sample a had the most hits and correspondingly lowest percentage overlap with samples b and c, likely due to the much higher number of mapped reads for that sample (see A). C) Total crosslink sites by sample and their overlap as in B but represented in bar graph form. D) Total genes that overlapped between biological replicates. The first column forms the basis for the curated, target gene set. E) Average crosslink sites per gene depending on overlap between biological replicates, showing that genes overlapping in a 3 biological replicates had a higher average number of crosslink sites predicted per gene. F) Enrichment of crosslink sites in the 3'UTRs of *T. gondii* genes in each of 3 biological replicates. G) Read mapping data across the ROCY1 locus for all three biological replicates, showing extensive mapping of reads across all exons in the gene for the input sample (blue) and enriched regions putatively bound by the ROCY1 protein shown in pink. Data available in Source Data File.

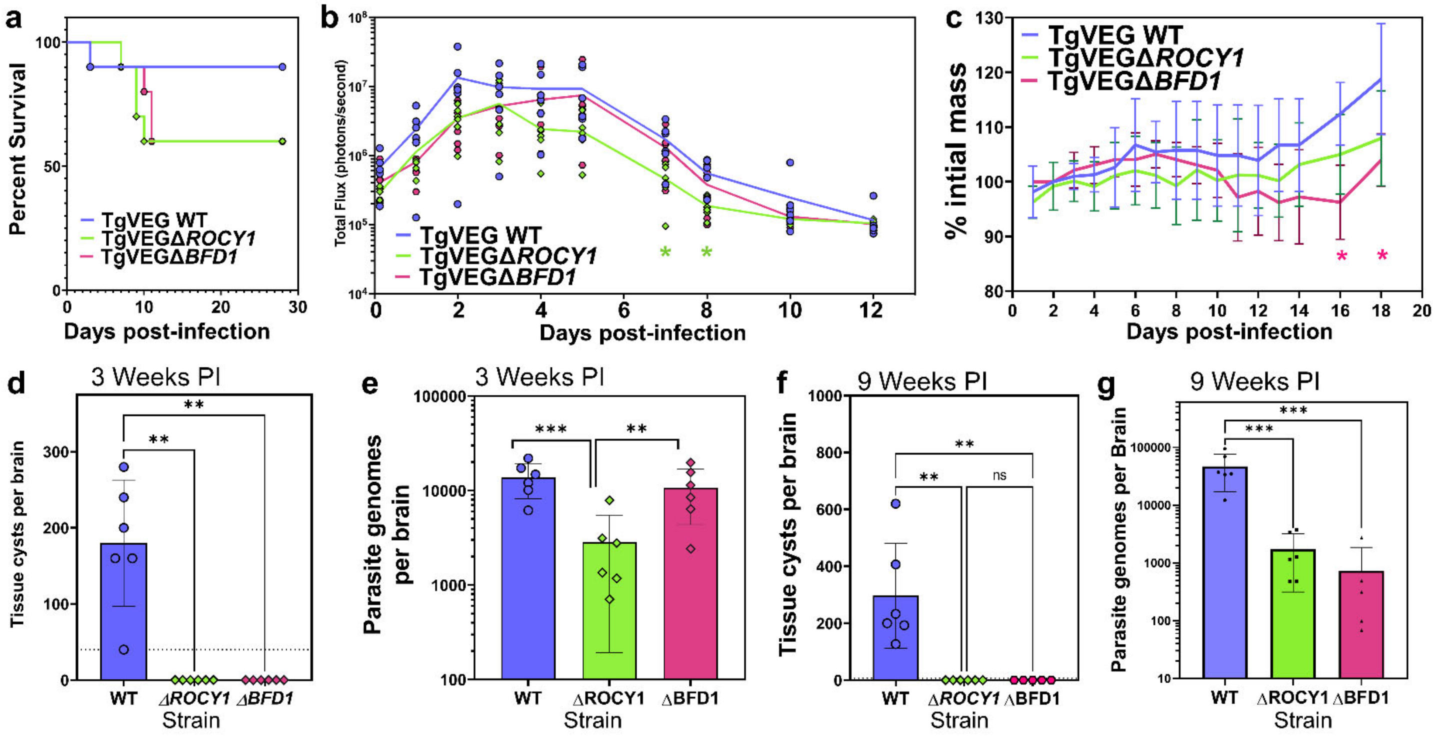

Figure S5: Data presented here and in Figure 7 represent two experiments with similar experimental regimens and data collection. Experiment 1 is mostly represented in Figure 7 and experiment 2 is mostly represented here int his supplementary figure. A) Mouse mortality over the course of acute infection with TgVEG wild type, TgVEGΔROCY1 or TgVEGΔBFD1 parasites for the experiment shown in **Figure 7** (Experiment 1; N=5 mice per infecting strain). While mortality rates were higher in mice infected with the knockout parasites this difference was not statistically significant (P=0.31 using the Log-rank test). B,C) *In vivo* BLI data quantifying parasite burden (B; median data shown) or mouse weight loss (C; mean +/- SD) over the course of the acute phase of infection with the indicated parasite strains in Experiment 2 (N=6 mice per infecting parasite strain). Asterisks indicate significant (P<0.05) differences in luminescent signal between knockouts and controls, and in this experiment the only strain and time points that were significantly different from wild type were for TgVEGΔROCY1 parasite burden on days 7 and 8 post-infection (*P*=0.03 and 0.02, respectively) and for TgVEGΔBFD1 mouse weight on days 16 and 18 post-infection (*P*=0.003 and 0.03, respectively). Both datasets were analyzed using 2 way ANOVA followed by Tukey's Multiple Comparison post-test. In contrast to the first experiment shown in **Figure 7** and mortality shown here for the first experiment in panel (A), no mice became morbid over the course of experiment 2. D,F) Quantification of cysts per brain in mice infected with the indicated strains at 3 (D; limit of detection = 40 cysts) and 9 (F; limit of detection = 7 cysts) weeks post-infection, showing a failure to detect cysts in any TgVEGΔROCY1 or TgVEGΔBFD1-infected mice at either time point (N=6 mice per strain for all conditions except for TgVEGΔBFD1 at 9 weeks PI where N=5). E,G) Quantitative PCR based estimate of the number of parasite genome equivalents per brain at 3 (E) and 9 (G) weeks post-infection. While all 3 strains could be detected in the brain of the infected mice (despite the lack of any detectable cysts in mice infected with ΔROCY1 or ΔBFD1), TgVEGΔROCY1-infected mice had significantly lower numbers of parasite genome equivalents compared to TgVEG WT-infected mice at 3 weeks PI. At 9 weeks PI, genome equivalents per brain for both ΔROCY1 and ΔBFD1 parasites were significantly lower than wild type, suggesting that ΔROCY1 and ΔBFD1 parasites may be cleared more effectively than wild type parasites. N=6 per strain except for ΔBFD1 at 9 weeks PI which had N=5 mice. **:P<0.01; ***: P<0.001. Statistical significance determined by one way ANOVA followed by Sidak's multiple comparison post-hoc test. Data available in Source Data File.

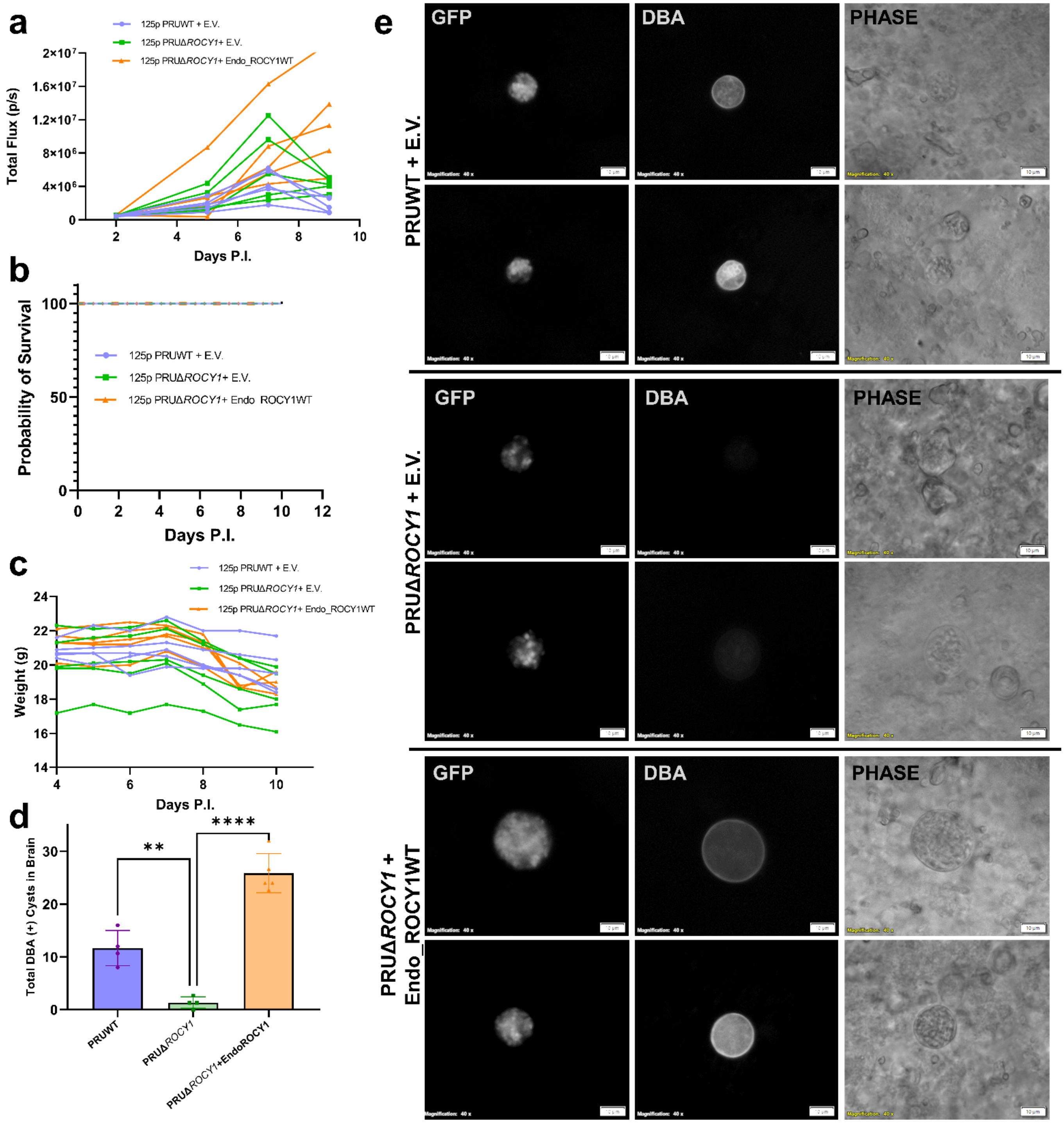

Figure S6: Mice were infected with 125 tachyzoites of TgPRU:WT, TgPRUΔ*ROCY1* and TgPRUΔ*ROCY1*:Endo_ROCY1 (N=5 per strain) to determine the impact of genetic complementation with ROCY1 expressed from its endogenous promoter. A) *In vivo* bioluminescence imaging of infected mice. B) Mouse survival rate was 100% at this dose for all strains. C) Mouse weight change over the course of the experiment. D) Quantification of brain cysts in surviving mice showing significantly reduced cyst numbers in TgPRUΔROCY1 compared to TgPRU:WT (**: P<0.01) and the significant complementation of this phenotype in TgPRUΔROCY1:Endo_ROCY1 (****: P<0.0001). Statistical significance determined by one way ANOVA followed by Sidak's Multiple comparison post-test. E) Representative images of tissue cysts from mice infected with 125 TgPRUWT, TgPRUΔ*ROCY1* or TgPRUΔ*ROCY1*:Endo_ROCY1 parasites and stained with DBA. GFP signal is derived from the parasites. A small number of cyst-like structures could be found in mice infected with PRUΔROCY1 but in many cases stained only weakly with DBA. Only those that stained fully with DBA were counted as DBA-positive cysts. Scale bar: 10 µm. This experiment was performed once. Data available in Source Data File.

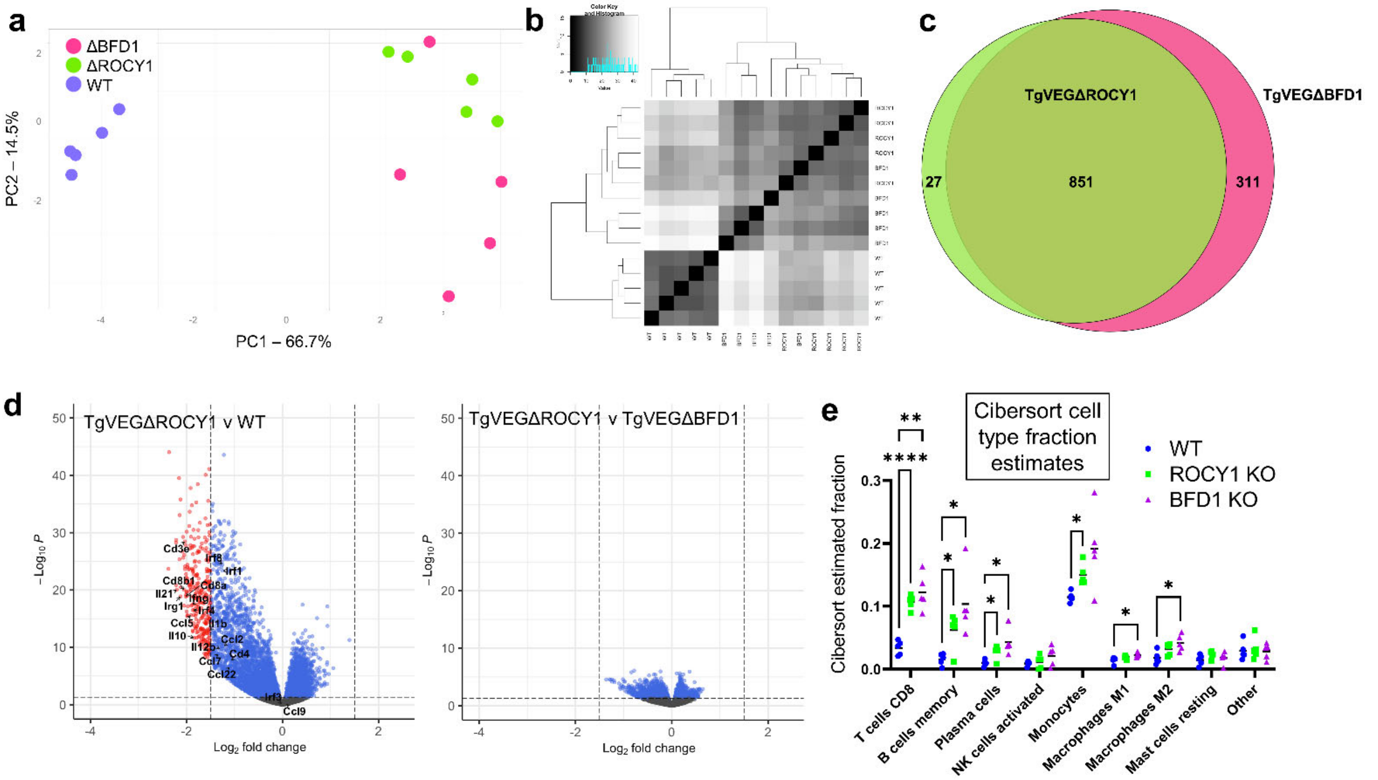

f

Gene sets enriched in TgVEGΔROCY1 *in vivo* mouse brain infection						
Rank	*Gene Set (GS)*	*GS size*	*Normalized enrichment score*	*Nominal p-value*	*FDR q-value*	*FWER p-value*
1	Hallmark_Complement	177	-1.90	0.000	0.039	0.017
2	Hallmark_Apoptosis	156	-1.89	0.000	0.022	0.021
3	Hallmark_IL2_STAT5_Signaling	192	-1.83	0.000	0.038	0.052
4	Hallmark_PI3K_AKT_mTOR_Signaling	102	-1.80	0.023	0.033	0.056
5	Hallmark_E2F_Targets	199	-1.80	0.000	0.027	0.056
6	Hallmark_G2M_Checkpoint	194	-1.80	0.000	0.023	0.059
7	Hallmark_Coagulation	102	-1.79	0.000	0.021	0.078
8	Hallmark_Allograft_Rejection	187	-1.76	0.000	0.024	0.081
9	Hallmark_Inflammatory_Response	183	-1.75	0.000	0.023	0.081
10	Hallmark_P53_Pathway	196	-1.75	0.010	0.022	0.093
11	Hallmark_KRAS_Signaling_UP	185	-1.74	0.000	0.026	0.115
12	Hallmark_TNFa_Signaling_via_NFKb	189	-1.72	0.000	0.033	0.118
13	Hallmark_Unfolded_Protein_Response	111	-1.70	0.000	0.035	0.118
14	Hallmark_Mitotic_Spindle	197	-1.70	0.010	0.034	0.118
15	Hallmark_Angiogenesis	31	-1.69	0.000	0.032	0.136
16	Hallmark_IL6_JAK_STAT3_Signaling	80	-1.65	0.000	0.039	0.137
17	Hallmark_UV_Response_UP	149	-1.64	0.016	0.042	0.164
18	Hallmark_MYC_Targets_V2	57	-1.61	0.004	0.050	0.166
19	Hallmark_Hypoxia	183	-1.61	0.044	0.049	0.194

g

Gene sets enriched in TgVEGΔBFD1 *in vivo* mouse brain infection						
Rank	*Gene Set (GS)*	*GS size*	*Normalized enrichment score*	*Nominal p-value*	*FDR q-value*	*FWER p-value*
1	Hallmark_Mitotic_Spindle	197	-1.78	0.000	0.011	0.008
2	Hallmark_G2M_Checkpoint	194	-1.74	0.000	0.014	0.021
3	Hallmark_IL2_STAT5_Signaling	192	-1.67	0.000	0.017	0.038
4	Hallmark_PI3K_AKT_mTOR_Signaling	102	-1.66	0.023	0.018	0.050
5	Hallmark_Allograft_Rejection	187	-1.64	0.000	0.018	0.063
6	Hallmark_E2F_Targets	199	-1.63	0.000	0.016	0.067
7	Hallmark_Reactive_Oxygen_Species_Pathway	48	-1.61	0.000	0.020	0.089
8	Hallmark_Inflammatory_Response	183	-1.61	0.000	0.018	0.089
9	Hallmark_Complement	177	-1.60	0.000	0.020	0.089
10	Hallmark_P53_Pathway	196	-1.59	0.025	0.022	0.099
11	Hallmark_Apoptosis	156	-1.56	0.000	0.027	0.104
12	Hallmark_TNFa_Signaling_via_NFKb	189	-1.54	0.000	0.037	0.176

h

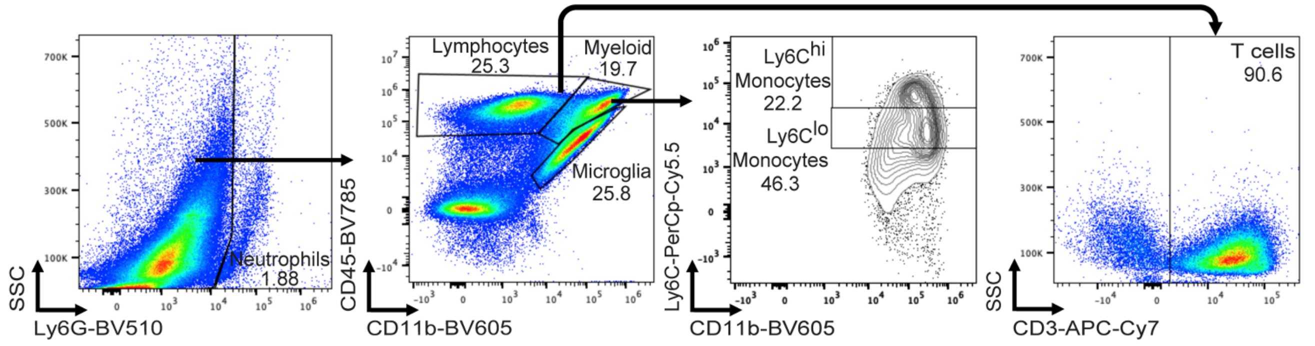

Figure S7. TgVEGΔBFD1 and TgVEGΔROCY1 infection initiates more inflammatory signaling in mouse brains as compared to TgVEG WT at 4 weeks post infection. All data derived from N=5 infected mice per parasite strain. A) Principal components (PC) 1 and 2 of sequenced RNA isolated 4 weeks post-infection from the brains of TgVEGΔBFD1, TgVEGΔROCY1, and TgVEG WT infected mice (data from this infection experiment are shown in **Figure 7**), showing a clear separation between WT-infected mouse brain

transcriptomes and those from mice infected with ΔROCY1 or ΔBFD1 parasites. N=5 infected mice per parasite strain. B) Unsupervised clustering of transcript abundance datasets isolated from the brains of TgVEGΔBFD1, TgVEGΔROCY1, and TgVEG WT infected mice, again showing the similarities between mouse brain transcriptomes from ΔROCY1 or ΔBFD1 infected mice compared to WT. C) Venn diagram of mouse genes with significant differences ($|\text{Log}_2$ Fold Change$| \geq 1$ and Padj < 0.05) in transcript abundance in knockout versus WT infections. D) Volcano plots of sequenced RNA isolated from mouse brains 4 weeks post infection comparing host transcriptional profiles between TgVEG WT and TgVEGΔROCY1 (left) and TgVEGΔROCY1 and TgVEGΔBFD1 (right). Red datapoints indicate genes with transcript abundances having $|\text{Log}_2$ fold difference$| > 1.5$ and Padj ≤ 0.05 for the indicated comparison. Some transcripts with known or putative immune function are labeled. E) Cibersort analysis of RNAseq data in (D) showing higher estimated proportions of CD8+ T cells as well as a other select cell types in mice infected with ΔROCY1 or ΔBFD1 parasites compared to controls. (***: P<0.001; **: P<0.01; *: P<0.05 based on one way ANOVA followed by Sidak's multiple comparison post-test. F, G) Gene set enrichment analysis showing "Hallmark" datasets significantly enriched in TgVEGΔROCY1 (F) or TgVEGΔBFD1- (G) infected mouse brains compared to TgVEG WT. All significantly enriched hallmark pathways in each knockout compared to wild type are shown, demonstrating significant overlap in the pathways that are differentially regulated between each knockout. H) Gating strategy for flow cytometry experiments on brains from mice infected with *T. gondii* VEG WT and ΔROCY1. Sample sizes for this experiment are in the main Figure panel figure legend. Data available in Source Data File.

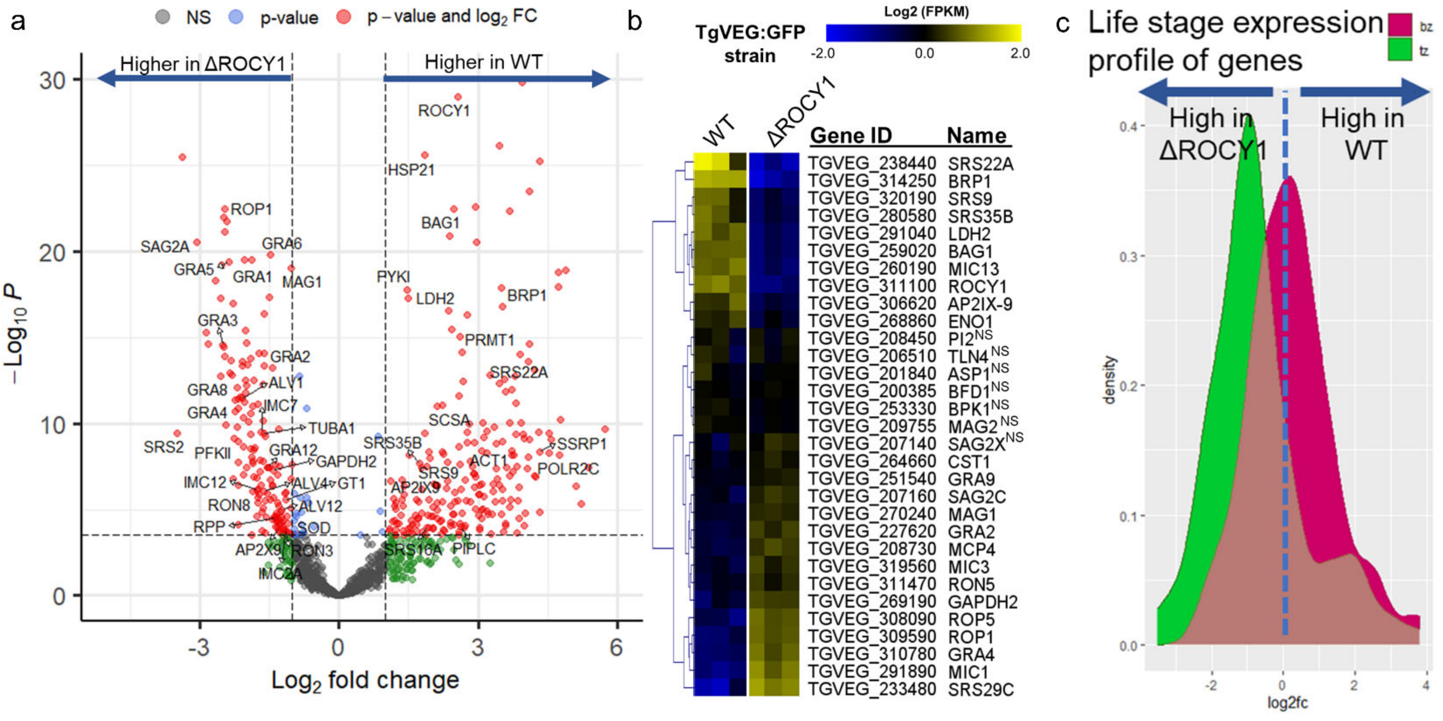

Figure S8: Transcriptomic analysis on brain-resident parasites during the chronic phase of infection with etiher wild type *T. gondii* VEG or TgVEGΔROCY1. Mice were infected with 100,000 parasites of TgVEG:WT or TgVEGΔROCY1 and brains were harveted on day 30 post-infection. After cell straining and syringe lysis to reduce contaminating host cell transcripts RNA was isolated (N=3 per parasite strain) and sequenced. After sequencing and mapping to the *T. gondii* VEG genome (version 43; www.toxodb.org) and then removing genes with low read counts across multiple samples, transcript abundance data from 1,225 genes were normalized and log-transformed based using DESeq2 and of these 492 had transcript abundance that significantly differed ($|\text{Log2FC}| > 1$ and Padj < 0.05). A) Volcano plot showing genes with significant (Padj<0.05; |log2FC > 1|) differences in transcript abundance where select genes are labeled with their gene name. Raw data can be found in Table S4. B) Heat map showing transcript abundance for representative bradyzoite and tachyozite genes in brain-resident parasites. Mean-centered data are shown and genes marked with superscript "NS" did not show significant differences (|Log2FC > 1; Padj < 0.05) in transcript abundance in

brain-resident parasites 30 days after infection between wild type and ΔROCY1 parasites. C) Log2 fold-differences in transcript abundance between TgVEGWT and TgVEGΔROCY1 were plotted in separate density plots depending on whether they have been categorized as a "tachyzoite" (tz)- or "bradyzoite" (bz)-specific transcript based on previously published work (Croken et al. 2014). The overall trend of tachyzoite-specific genes having higher transcript abundance in TgVEGΔROCY1 parasites compared to TgVEGWT can be seen in the "left-shifted" plot of tz genes compared to bz genes. Densities were significantly different based on a two-sided Kolmogorov-Smirnov test (P=1.6x10^{-8}). Experiment was performed once. Data Available in Source Data File.

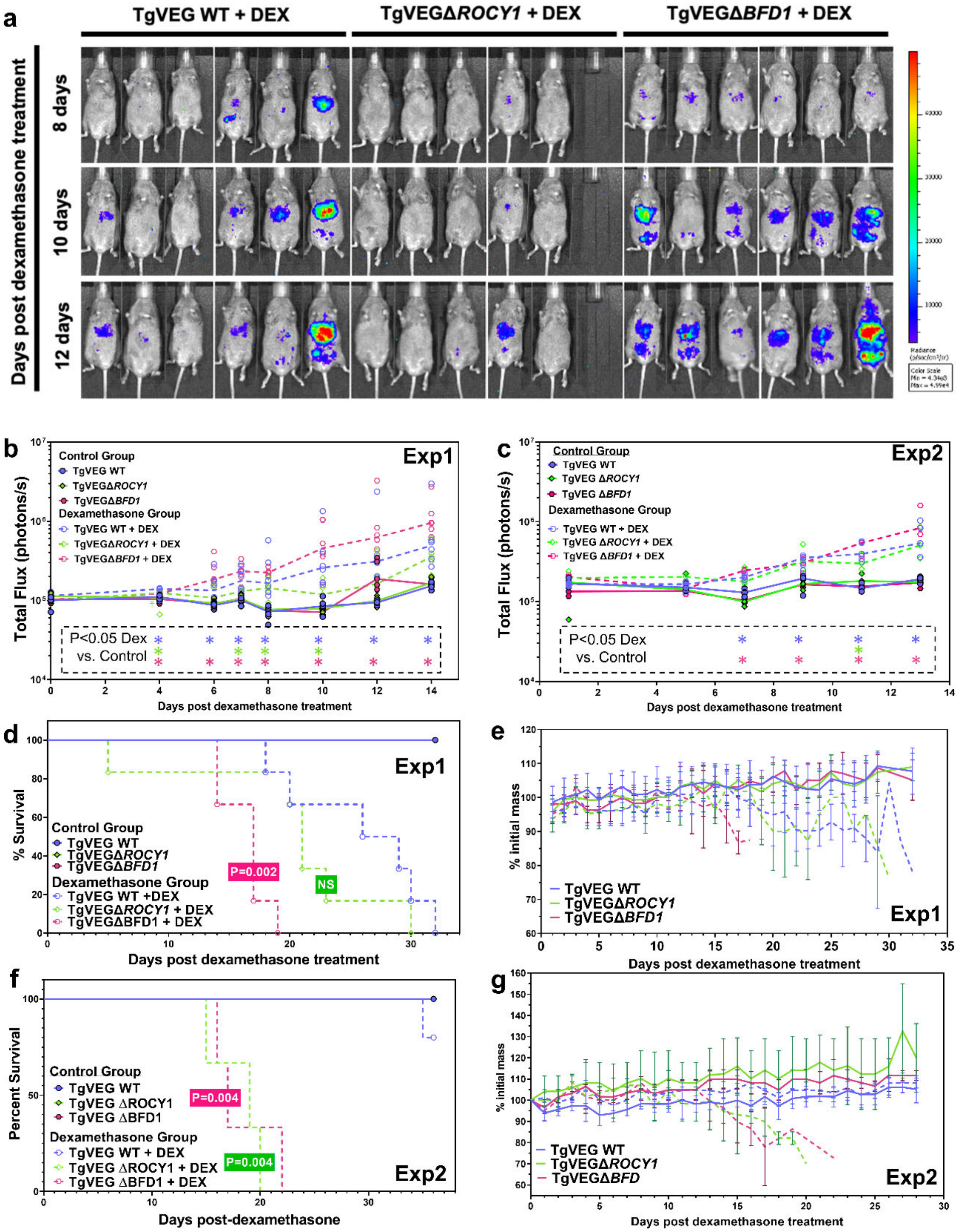

Figure S9: A) Select bioluminescent images showing parasite-derived luciferase signal in CBA/J mice infected with TgVEG WT, TgVEGΔ*ROCY1,* and TgVEGΔ*BFD1* parasites and treated with dexamethasone (20mg/L) provided in their drinking water beginning at 30 dpi. B) Quantification of bioluminescent imaging of mice infected

with TgVEG WT, TgVEGΔ*ROCY1,* and TgVEGΔ*BFD1* parasite belonging to either the control group (N=6 per parasite strain except ΔBFD1 where N=5) or the dexamethasone treatment group (N=6 per parasite strain). Each point represent an individual mouse and the line represents the mean. The double cross symbol (†) represents death due to handling/anesthesia. Dashed line annotations represent dexamethasone group compared to control group for each parasite strain. Blue annotations represent TgVEG WT:GFP-LUC, green annotations represent TgVEGΔ*ROCY1*:GFP-LUC, and pink annotations represent TgVEGΔ*BFD1*:GFP-LUC. B) Quantification and statistical comparisons of bioluminescent imaging of mice for Exp 1 infected with TgVEG WT, TgVEGΔ*ROCY1,* and TgVEGΔ*BFD1* treated with dexamethasone at 30 dpi. Each point represents an individual mouse and the line represents the median. Data from control group is represented by a solid line and dashed lines indicate data from the dexamethasone-treated group. Asterisks indicate significant (P<0.05) differences in bioluminescent signal at that timepoint comparing the control group to the dexamethasone-treatment group for each parasite strain using a Two-way ANOVA and Fisher's Exact LSD test. Only pre-planned comparisons were performed to minimize Type I Error. C) Quantification and statistical comparisons of bioluminescent imaging of mice for Exp 2 infected with TgVEG WT (N=5 for Dex, 4 for control), TgVEGΔ*ROCY1* N=3 for both conditions), and TgVEGΔ*BFD1* (N=3 for both) treated or not with dexamethasone at 30 dpi. Each point represents an individual mouse and the line represents the median. Data from control group is represented by a solid line and dashed lines indicate data from the dexamethasone-treated group. Asterisks indicate significant (P<0.05) differences in bioluminescent signal at that timepoint comparing the control group to the dexamethasone-treatment group for each parasite strain using a Two-way ANOVA and Fisher's Exact LSD test. Only pre-planned comparisons were performed to minimize Type I Error. D) Morbidity for mice in Experiment 1. Mice infected with TgVEGΔ*BFD1* succumbed significantly faster (P=0.002 based on Log-Rank Mantel-Cox Test) than those infected with TgVEG WT. E) Weight over the course of Experiment 1. F) Morbidity for mice in Experiment 2. Mice infected with both ΔROCY1 and ΔBFD1 succumbed significantly more rapidly to reactivation-derived pathogenesis compared to mice infected with wild type parasites (based on the Log-Rank Mantel-Cox test). G) Weight over the course of Experiment 2. Data available in Source Data File.
