# Supplementary material for: A transcriptional network required for bradyzoite development in Toxoplasma gondii is dispensable for recrudescent disease
Source: Nat Commun. 2023 Sep 28;14:6078. doi: 10.1038/s41467-023-40948-w (PMC10539341; doi:10.1038/s41467-023-40948-w)
Supplement: Supplementary file 3 — Description of additional supplementary files [file 41467_2023_40948_MOESM3_ESM.pdf]

Description of Additional Supplementary Files

File Name: Supplementary Data 1
Description: Primers used in the present study.

File Name: Supplementary Data 2
Description: Bulk RNAseq data from in vitro analysis of the effect of bradyzoite induction conditions on transcript abundance in wild type, ΔROCY1 and ΔBFD1 parasites in the *T. gondii* VEG genetic background.

File Name: Supplementary Data 3
Description: Summary data from enhanced crosslinking immunoprecipitation of FLAG-tagged ROCY1 and its putative target RNA substrates.

File Name: Supplementary Data 4
Description: RNAseq data of host and parasite transcript abundance from mice infected for ~30 days with wild type, ΔROCY1 or ΔBFD1 (for host gene expression) or wild type and ΔROCY1 (for parasite gene expression). RNA was taken from the brains of infected mice and immediately subjected to RNAseq analysis or after enriching for parasites by syringe lysis and washing.
