# Supplementary material for: A transcriptional network required for bradyzoite development in Toxoplasma gondii is dispensable for recrudescent disease
Source: Nat Commun. 2023 Sep 28;14:6078. doi: 10.1038/s41467-023-40948-w (PMC10539341; doi:10.1038/s41467-023-40948-w)
Supplement: Supplementary file 8 — Reporting Summary [file 41467_2023_40948_MOESM8_ESM.pdf]

nature portfolio

Corresponding author(s): Boyle, Jon P

Last updated by author(s): Jun 7, 2023

Reporting Summary

Nature Portfolio wishes to improve the reproducibility of the work that we publish. This form provides structure for consistency and transparency in reporting. For further information on Nature Portfolio policies, see our Editorial Policies and the Editorial Policy Checklist.

Statistics

For all statistical analyses, confirm that the following items are present in the figure legend, table legend, main text, or Methods section.

n/a	Confirmed	
☐	☒	The exact sample size (*n*) for each experimental group/condition, given as a discrete number and unit of measurement
☐	☒	A statement on whether measurements were taken from distinct samples or whether the same sample was measured repeatedly
☐	☒	The statistical test(s) used AND whether they are one- or two-sided<br>*Only common tests should be described solely by name; describe more complex techniques in the Methods section.*
☒	☐	A description of all covariates tested
☐	☒	A description of any assumptions or corrections, such as tests of normality and adjustment for multiple comparisons
☐	☒	A full description of the statistical parameters including central tendency (e.g. means) or other basic estimates (e.g. regression coefficient) AND variation (e.g. standard deviation) or associated estimates of uncertainty (e.g. confidence intervals)
☐	☒	For null hypothesis testing, the test statistic (e.g. *F*, *t*, *r*) with confidence intervals, effect sizes, degrees of freedom and *P* value noted<br>*Give P values as exact values whenever suitable.*
☒	☐	For Bayesian analysis, information on the choice of priors and Markov chain Monte Carlo settings
☒	☐	For hierarchical and complex designs, identification of the appropriate level for tests and full reporting of outcomes
☒	☐	Estimates of effect sizes (e.g. Cohen's *d*, Pearson's *r*), indicating how they were calculated

Our web collection on statistics for biologists contains articles on many of the points above.

Software and code

Policy information about availability of computer code

Data collection	Knime Analytics Platform 4.5.1; CellSens Dimension Imaging Software 1.16; FloJo Software 10.8.1.
Data analysis	CLC Genomics Workbench 22.0.1; TM4 MeViewer 4.9.0; Bowtie2 2.3.5.1; Samtools 1.9-66; FeatureCounts v2.0.0; Graphpad Prism 9.1; CutAdapt v4.0; FloJo Software 10.8.1.

For manuscripts utilizing custom algorithms or software that are central to the research but not yet described in published literature, software must be made available to editors and reviewers. We strongly encourage code deposition in a community repository (e.g. GitHub). See the Nature Portfolio guidelines for submitting code & software for further information.

Data

Policy information about availability of data

All manuscripts must include a data availability statement. This statement should provide the following information, where applicable:

- Accession codes, unique identifiers, or web links for publicly available datasets
- A description of any restrictions on data availability
- For clinical datasets or third party data, please ensure that the statement adheres to our policy

All RNAseq data have been deposited in the NCBI Gene Expression Omnibus and are publicly available. Accession numbers with full links are provided.

Field-specific reporting

Please select the one below that is the best fit for your research. If you are not sure, read the appropriate sections before making your selection.

☒ Life sciences ☐ Behavioural & social sciences ☐ Ecological, evolutionary & environmental sciences

For a reference copy of the document with all sections, see nature.com/documents/nr-reporting-summary-flat.pdf

Life sciences study design

All studies must disclose on these points even when the disclosure is negative.

Sample size	Sample size is determined based on the "experimental unit" concept, in other words the smallest unit to which a given treatment is applied. For example in bradyzoite switch assays the experimental unit is the coverslip that is treated with the control or high pH medium. In cases where data are taken from individual vacuoles (for example in measuring DBA staining intensity) each vacuole is considered the experimental unit. To determine apprpriate sample sizes for in vitro and in vivo experiments we relied on our previous experience in experimental design, where N=3 is sufficient for in in vitro assays for cyst formation (Sokol et al., eLife 2018) and N=5-6 for in vivo cyst quantification experiments (English et al., mSphere 2018).
Data exclusions	In certain cases mice that died before the end of the experiment were excluded from repeated measures analyses of mouse weight and in vivo bioluminescence signal. This was in order to allow for a 2 way ANOVA with repeated measures analysis to be used which is unable to account for missing data.
Replication	In vivo experiments were repeated at least twice and most of the results were consistent between experiments. However in some cases there were slight differences in the results (for example the time points at which there were significant differences between strains or the strains that showed significant differences) and we address these directly in the figure legends and results. For most in vitro experiments we performed it only a single time, but with sufficient independent biological replicates to estimate the mean accurately.
Randomization	Mice were shipped together and randomly assigned to different cages prior to experimentation. Randomization was not necessary for in vitro experiments as all host cells were derived from the same passage. Plates were all incubated in the same incubators under the same conditions, and therefore randomization would have provided more opportunity for experimental error while not having any direct impact on the outcome.
Blinding	In some cases (for example in screening T. gondii VEG knockouts for cyst formation defects) the person observing the slides was unaware of the strain/treatment combination when determining the percent cyst formation across multiple fields of view. However we did not use blinding for follow up experiments. Blinding requires dependence on others besides the experimenter to blind/unblind and this adds potential for experimental error/loss of the "answer key" and so for follow up experiments we did not blind the cyst induction experiments.

Reporting for specific materials, systems and methods

We require information from authors about some types of materials, experimental systems and methods used in many studies. Here, indicate whether each material, system or method listed is relevant to your study. If you are not sure if a list item applies to your research, read the appropriate section before selecting a response.

Materials & experimental systems

n/a	Involved in the study	
☐	☒	Antibodies
☐	☒	Eukaryotic cell lines
☒	☐	Palaeontology and archaeology
☐	☒	Animals and other organisms
☒	☐	Human research participants
☒	☐	Clinical data
☒	☐	Dual use research of concern

Methods

n/a	Involved in the study	
☒	☐	ChIP-seq
☐	☒	Flow cytometry
☒	☐	MRI-based neuroimaging

Antibodies

Antibodies used	Rhodamine-labeled Dolichos bifluorus agglutinin: Vector Labs, RL1032-2, Biotinylated Dolichos bifluorus agglutinin: Vector Labs B-1035-5, Rabbit anti-GFP antibody: Abcam ab290; Mouse anti Toxoplasma gondii MAF1b.
Validation	Dolichos lectins were validated by staining T. gondii cysts harvested from mouse brains. Staining of the cyst wall surrounding the enclosed bradyzoites was used to validate the reagent and to determine the optimal dilution. Maf1b staining was consistently verified to be in the parasitophorous vacuole. Mouse anti-GFP antibody is validated by the manufacturer (Abcam) and was found to be a rabbit primary antibody based on our effective use of anit Rabbit secondary antibodies during the IHC assay.

Eukaryotic cell lines

Policy information about cell lines

Cell line source(s)	Human foreskin fibroblasts: These were harvested from de-identified foreskin tissue obtained in 2007 from the Stanford University Hospital.
Authentication	Human foreskin fibroblasts have a characteristic shape and also contact inhibit. These properties are used to validate them each time they are passed and grown.
Mycoplasma contamination	HFF cell lines are tested monthly as are parasite lines grown in the laboratory using a PCR test.
Commonly misidentified lines (See ICLAC register)	Not applicable

Animals and other organisms

Policy information about studies involving animals; ARRIVE guidelines recommended for reporting animal research

Laboratory animals	Strains: Balb/c, CBA/J. Age: 6-10 weeks. Sex: Female
Wild animals	The study did not involve wild animals.
Field-collected samples	No samples were collected from the field.
Ethics oversight	All procedures involving animals were approved by the local IACUC at either the University of Pittsburgh or the University of California Irvine. Laboratory safety and recombinant DNA procedures were approved by the local Environmental Health and Safety and Institutional Biosafety Committees at the University of Pittsburgh, Indiana University School of Medicine, and University of California Irvine. For animal experiments female mice were used in all studies as this is the most well-characterized animal model for T. gondii infection and our goal was to examine cyst formation of our mutants rather than directly assess the impact on the host. It would be, in this case, ethically questionable to perform all experiments in males and females as this would require additional pilot studies in males and double the number of animals used in our experiments unnecessarily.

Note that full information on the approval of the study protocol must also be provided in the manuscript.

Flow Cytometry

Plots

Confirm that:

☒ The axis labels state the marker and fluorochrome used (e.g. CD4-FITC).

☒ The axis scales are clearly visible. Include numbers along axes only for bottom left plot of group (a 'group' is an analysis of identical markers).

☒ All plots are contour plots with outliers or pseudocolor plots.

☒ A numerical value for number of cells or percentage (with statistics) is provided.

Methodology

Sample preparation	For flow cytometric analyses, C57BL/6 mice were infected with 200 TgVEG WT-GFP-LUC or TgVEGΔROCY1-GFP-LUC parasites in 200 µL of PBS via intraperitoneal injection. At day 28 post-infection, mice were transcardially perfused with 50 mL of 1X PBS to remove non-adherent blood cells. Brains were harvested and digested using Dispase II diluted in Hepes-buffered saline. To remove myelin, 35% and 75% percoll gradients were used. To block non-specific binding of antibodies to immune cells, the isolated cells were resuspended in 10% TrueStain FcX Buffer in staining buffer (3% fetal bovine serum in 1X PBS). Cells were surface-stained with fluorescent dye-conjugated antibodies diluted in staining buffer. Cells were then resuspended in 1X PBS and run on the Novocyte flow cytometer.
Instrument	Samples were run on the Agilent Novocyte flow cytometer.
Software	Flow cytometry data were analyzed and graphically represented utilizing FlowJo software.
Cell population abundance	Immune cells comprised an average of approximately 80% of the isolated singlets.
Gating strategy	To define singlets, cells were gated on forward scatter height and forward scatter area. From these singlets, neutrophils were gated out using Ly6G. From the Ly6G- population, infiltrating cells were defined as CD11b+CD45+, microglia were defined as CD11b+ CD45 intermediate, and lymphocytes were defined as CD11b-CD45+. From the infiltrating myeloid population, inflammatory monocytes were defined as Ly6C high and patrolling monocytes were defined as Ly6C low. From the lymphocyte population, T cells were defined as CD3+.

☒ Tick this box to confirm that a figure exemplifying the gating strategy is provided in the Supplementary Information.
